# Evaluating the Effect of Sex on Mortality Risks in Medieval Ireland

**DOI:** 10.1002/ajpa.70040

**Published:** 2025-04-09

**Authors:** Allison C. Ham

**Affiliations:** ^1^ Department of Anthropology Penn State University University Park Pennsylvania USA

**Keywords:** bioarchaeology, female survival advantage, hazards models, sex differentials in mortality

## Abstract

**Objectives:**

This study evaluates the effect of sex on mortality risks in medieval Ireland to advance our understanding of the social, biological, and environmental factors that were deleterious to female health and survival in the past.

**Materials and Methods:**

Data on age‐at‐death and sex was collected on 335 skeletonized individuals from 10 archaeological sites dating to the early medieval (500–1150 ce) and late medieval (1150–1550 ce) periods in Ireland. Transition analysis (TA2) was used to estimate age‐at‐death for all individuals with visibly fused pelvic and long bone epiphyses. For all other individuals, age‐at‐death was estimated using dental development and epiphyseal fusion. Morphological traits of the pelvis and cranium and metric measurements were used to estimate sex. A Gompertz‐Makeham hazards model with a proportional hazards specification was used to examine the effect of the sex covariate on mortality risks.

**Results:**

The Gompertz‐Makeham hazards model failed to reveal an effect of sex on mortality risks in this context. No significant temporal variation in the effect of sex on the model was observed across sites.

**Conclusions:**

The results failed to find an effect of the sex covariate on the mortality profile using hazards analysis. However, the similar mortality profiles observed between medieval Irish males and females could reflect cultural barriers and/or differential environmental exposures that counteracted the innate female survival advantage observed today.

## 1 Introduction

Over the last 150 years, a pronounced sex gap in longevity has emerged as human life expectancy has steadily risen across the globe (Oeppen and Vaupel 2002; Wilmoth 2000). Modern females experience lower age‐specific mortality risks compared to males beginning in utero and live 5 years longer on average, but this figure is as high as 10–15 years in some countries (Austad 2006; Barford et al. 2006; Dattani et al. 2023). Females may possess an innate survival advantage due to several genetic and hormonal factors (Austad and Bartke 2015; Schurz et al. 2019), but context‐specific practices and gendered behavior also influence sex differentials in mortality via exposure to life‐threatening conditions and scenarios (Chen et al. 1981). Thus, establishing long‐term trends in sex differentials in mortality is critical to understanding how large‐scale ecological and cultural shifts lead to changes in human behavior, which intersect with sex‐related biological processes to disproportionately affect the health of certain groups. However, our knowledge is limited by the reliability of historic demographic data (e.g., parish registers, civil registration records) and the underreporting and/or absence of females in the written record. Hence, the use of bioarchaeological data from diverse contexts can advance our knowledge of sex differentials in mortality and help discern how unique temporal, environmental, and cultural settings modulated the innate female survival advantage. To this end, this study uses hazards analysis to examine how social, biological, and environmental factors in medieval Ireland could have interacted to produce a female bias in mortality risks in this context.

Efforts to explain the modern female bias in longevity commonly rely on identifying the biological mechanisms related to sex‐specific mortality risks that bestow an innate survival advantage on females. For instance, a male bias in neonate and infant mortality (0–1 years) is common due to sex‐linked genetic factors that produce differences in fetal growth/development and immuno‐response (Chlamydas et al. 2022; Laube and Thome 2022; Muenchhoff and Goulder 2014; Schurz et al. 2019; Townsel et al. 2017; Waldron 1998). Moreover, males generally have increased susceptibility to most parasitic, bacterial, and viral infections (with a few exceptions) (Chlamydas et al. 2022; Muenchhoff and Goulder 2014). Several processes related to human sexual variation have been linked to increased male susceptibility to infection and life‐threatening chronic diseases (Case and Paxson 2005; Guerra‐Silveira and Abad‐Franch 2013; Muenchhoff and Goulder 2014; van Lunzen and Altfeld 2014), including the role of steroid hormones in the immune and endocrine systems and the lack of a second X chromosome in typical males (Austad and Bartke 2015; Christensen et al. 2000; Klein 2000; Migeon 2008; Pennell et al. 2012; Schurz et al. 2019). Typical females possess two X chromosomes and are functional mosaics for X‐linked genes (i.e., they receive a X chromosome from each parent with non‐identical gene content). This reduces the likelihood of disease expression and contributes to a female immunological advantage over males (Migeon 2007, 2008). These sex biases in infection also relate to a stronger Th1 (T‐helper type 1 cell) immune response enhanced by higher levels of estrogen in females. However, higher levels of proinflammatory immunity can lead to increased immunopathology in some infections and a predisposition to autoimmune diseases in females (Xing et al. 2022). Additionally, age‐related hormonal fluctuations over the female life course (e.g., puberty, pregnancy, menopause) modulate the immune system and can increase disease susceptibility and severity (Han et al. 2021; Kourtis et al. 2014; Oertelt‐Prigione 2012). Sex‐specific biological mechanisms play a key role in the production of an innate female survival advantage, but context‐specific external pressures also help to shape sex‐specific mortality risks.

Exceptions to the female survival advantage are credited to context‐specific cultural and behavioral heterogeneity and environmental variability that increase female exposure to life‐threatening conditions and/or scenarios (Chen et al. 1981; Coale 1991; Schacht et al. 2020; Teriokhin et al. 2004; Wall 1981). In patriarchal societies (i.e., male‐dominated social arrangements of power observed at both the familial and institutional‐level (Ortner 2022)), females are traditionally treated as subordinates, lack autonomy, and have poorer access to medical care, educational resources, and economic opportunities (Pennington et al. 2023). Moreover, expectations of reproductive behavior in these settings are detrimental to female survival, due to the positive association between high fertility and maternal mortality (Girum and Wasie 2017; Jain 2011; Kamel 1984; Zureick‐Brown et al. 2013). Patriarchal societies with strongly enforced gender norms (i.e., the physical, biological, psychological, and behavioral traits ascribed to a given sex (Zuckerman and Crandall 2019)) and sex‐based inequalities are found to have higher recorded levels of excess female mortality in both historic and modern communities (Beltrán Tapia and Gallego‐Martínez 2017; Bolund et al. 2016; Bongaarts and Guilmoto 2015; Hsu et al. 2021; Klasen 1998; Pennington et al. 2023; Salinari et al. 2022).

In the late 19th century, a modest female survival advantage of ~1–3 years existed in several Western contexts, but not across all age groups. Females generally had higher levels of mortality in late childhood, adolescence, and reproductive adulthood years (Glei and Horiuchi 2007; Horiuchi 1999; McNay et al. 2005; Perner et al. 2022; Tabutin and Willems 1998). Excess female mortality in adulthood during the late 19th century could relate to higher female reproductive costs. This is supported by the connection between fluctuating fertility and maternal mortality rates during this period and the subsequent decline in excess mortality for reproductive‐aged females in the early 20th century (Albanesi and Olivetti 2016; Bolund et al. 2016). However, these sex‐specific reproductive factors do not fully explain the emergence of lower age‐specific mortality risks for females across all age categories in Western industrialized societies during this period (Goldin and Lleras‐Muney 2019; Retherford 1975). Increased pathogen exposure and susceptibility to specific infections (e.g., tuberculosis) also helped to generate excess female mortality in the abovementioned age groups, which contrasts with the male bias in infection observed today. Several explanations have been proposed to explain this elevated vulnerability to infectious disease in females, including the traditional responsibilities of women in childcare and caregiving, the inferior social and economic position of women, and increased immunopathology due to pregnancy (Chan and Smith 2018; Goldin and Lleras‐Muney 2019; Kourtis et al. 2014; McNay et al. 2005). Subsequently, the reduction in the infectious disease burden combined with the decline in fertility during the early 20th century had a pronounced effect on female age‐specific mortality risks.

Unfortunately, our knowledge of pre‐industrial trends in sex differentials in mortality is more limited due to the earliest national population data dating to the mid‐18th century (Willigan and Lynch 1982; Wilmoth 2002). It is hypothesized that the large‐scale changes associated with the Neolithic transition (e.g., shifts in subsistence and settlement patterns) had a stronger effect on female mortality compared to males, in large part due to their effect on fertility rates, gendered domestic labor roles, and risks of pathogen exposure (Boldsen and Paine 1995). For example, the nutritional changes associated with the shift from foraging to farming are hypothesized to have led to increased female reproductive capability and decreased interbirth intervals, producing higher fertility rates and a decline in female survival (Bentley et al. 2001; Boldsen and Paine 1995). In essence, the transition to agriculture created social and biological circumstances that had a disproportionate effect on female mortality. However, the reconstruction of paleodemographic trends from pre‐industrial contexts using skeletonized individuals faces several methodological challenges, including small sample sizes, the selective bias of archaeological cemeteries, and the misestimation of age‐at‐death (to name a few) (Boldsen et al. 2022; Wood et al. 1992). The incorporation of hazards analysis (e.g., Gompertz, Gompertz‐Makeham, Siler mortality models) into bioarchaeological research helps to overcome several of these challenges and has successfully been used to reveal sex differences in mortality risks within pre‐industrial contexts in both attritional and catastrophic mortality settings (DeWitte 2010; DeWitte and Yaussy 2020; Fojas 2022; Godde et al. 2020; Griffin et al. 2018; Wilson 2014). Several of these studies provide support for a female mortality disadvantage in pre‐industrial contexts, particularly during reproductive years (Fojas 2022; Wilson 2014). However, no consistent trend in sex differentials in mortality has been established across pre‐industrial settings. This could reflect context variability in environmental, economic, and social circumstances or the methodological challenges mentioned above.

Overall, the emergence of the female bias in longevity in the early 20th century was a product of the widespread socioeconomic reforms and improvements in environmental and living conditions that had a disproportionate effect on age‐specific female mortality risks (Goldin and Lleras‐Muney 2019; Vaupel et al. 2021; Wilmoth 2000; Zarulli et al. 2021). Under the principle of uniformitarianism, it is presumed that the biological mechanisms connected to the innate female survival advantage observed today were operating in a similar manner in the past. Subsequently, any deviations from this trend could suggest the presence of cultural barriers and/or differential exposure to environmental pressures that were deleterious to female health and survival. Thus, by establishing trends in sex differentials in mortality, bioarchaeologists can advance our understanding of how cultural and environmental shifts disproportionately impacted female health and mortality outcomes across diverse contexts in the past.

### 1.1 Medieval Ireland

Hazards analysis has successfully been used to explore sex differences in mortality risks from a bioarchaeological perspective in several locations (e.g., England (DeWitte 2010; Godde et al. 2020) and North America (Fojas 2022; Wilson 2014)), but the topic remains largely unaddressed within medieval Ireland (500–1550 ce), which represents an ideal context due to its rich documentary and archaeological record. The early medieval period (500–1150 ce) has a rich source of historical texts, including chronicles, genealogies, and legal tracts, that provide valuable insight into Irish society (Bitel 1996; Kelly 1988; Oxenham 2016). The Anglo‐Norman invasion (c. 1169–1170 ce) marked the beginning of the late medieval period in Ireland and brought about drastic socioeconomic and political changes, particularly to the southeast and eastern regions. Prior bioarchaeological assessments of sex differences in age‐at‐death distributions from medieval Ireland have revealed no consistent pattern across archaeological sites (McKenzie and Murphy 2018; Novak 2015; Novak et al. 2017; Power 1995, 1997; Scott 2006). However, there is evidence to suggest temporal variation in the age‐at‐death distribution with mortality increasing for females in the late medieval period (Power 1997). Given the aforementioned evidence for a female mortality disadvantage in other pre‐industrial settings, it is expected that sex will have a significant effect on mortality risks and females will have higher hazards of death compared to males. Moreover, it is expected that the effect of sex on mortality risks will be more pronounced in the late medieval versus the early medieval period, with female mortality risks increasing during the former.

## 2 Materials and Methods

**FIGURE 1:**
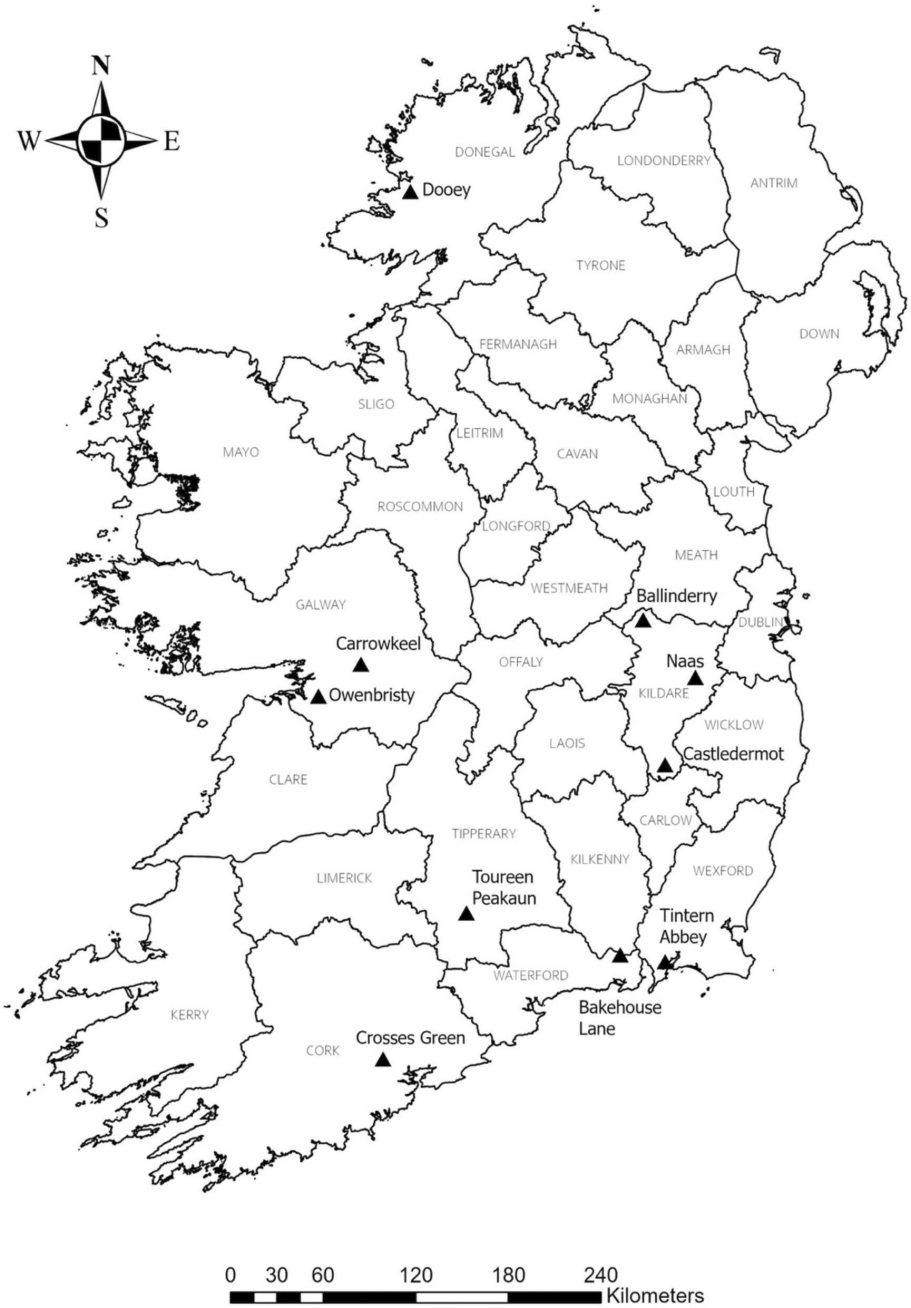
Map of medieval Irish sites included in the study.

The 10 archaeological sites included in this study date to the early medieval (500–1150 ce) and late medieval (1150–1550 ce) periods in Ireland (Table 1). The location of the sites is depicted in Figure 1. The sites were previously dated using a variety of evidence, including radiocarbon dating, artifact assemblages, and stratigraphic information, and have been described elsewhere (Barry et al. 1997; Geber 2011; Halpin 2001; Harte and Richardson 2010; Hurley and Sheehan 1995; Ó Carragáin 2017; Ó Donnabháin 2010; Ó Ríordáin and Rynne 1961; Tesorieri 2012; Wilkins and Lalonde 2008). Skeletal data on age‐at‐death and sex was collected from 335 skeletonized individuals excavated at these sites. All skeletonized individuals analyzed in this study are housed at the University of College Cork, Ireland (UCC) with the permission of the National Museum of Ireland (NMI). Additionally, all skeletonized individuals were excavated with an archaeological license, as required by Section 26 of the Irish National Monuments Act of 1930, and permission to undertake this study was given by the UCC Department of Archaeology and a NMI Board Member. The NMI supports the study of skeletonized individuals from archaeological contexts to advance knowledge of past Irish peoples (see Human Remains Policy 2019). All individuals were treated ethically and with the utmost care and respect during skeletal data collection and analysis.

**TABLE 1:** List of archaeological sites with their excavation license numbers, time period, date range, and number of male and female individuals included in the study.

Site name	Time period	Date range (ce)	Female	Male	Total
Dooey (E33)	Early medieval	6th–8th centuries	26	21	47
Owenbristy (E3770)	Early medieval	6th–10th centuries	12	22	34
Carrowkeel (A02411)	Early medieval	7th–11th centuries	7	9	16
Toureen Peakaun (E0247)	Late medieval	7th–12th centuries	0	2	2
Bakehouse Lane (E435)	Late medieval	12th–16th centuries	19	16	35
Naas (E0094)	Late medieval	13th–14th centuries	3	7	10
Ballinderry (E1638)	Late medieval	13th–14th centuries	38	29	67
Tintern Abbey (E237, E0075)	Late medieval	13th–16th centuries	17	25	42
Crosse's Green (E0103)	Late medieval	13th–16th centuries	35	37	72
Castledermot (E0750)	Late medieval	14th–16th centuries	4	6	10
Total			161	174	335

### 2.1 Age Estimation

To avoid issues with traditional phase‐based age estimation methods (see Boldsen et al. 2022; Getz 2020), this study used transition analysis, as described by Boldsen et al. (2002), to estimate age‐at‐death for all individuals with visibly fused pelvic and long bone epiphyses. This form of transition analysis employs logit regression to calculate the conditional probability that a skeletonized individual will display indicators for age‐specific stages for multiple age‐related morphological traits given age $\left(\mathrm{Pr}\left(\;c_{j}|a\right)\right)$ using a known age reference sample. The posterior probability that an individual from the target cemetery died at a certain age based on the observed age indicator stages $\left(\mathrm{Pr}\left(a|c_{j}\right)\right)$ is derived using Bayes' Theorem. The Anthropological Database, Odense University (ADBOU) Age Estimation software (TA2) was used to generate point estimates and confidence intervals for age‐at‐death with the archaeological informative prior distribution selected. The archaeological informative prior is derived from 17th century Danish parish records that represent a generalized pre‐industrial mortality profile. It is the most appropriate prior selection for this study, due to the absence of a population‐specific age‐at‐death distribution (Bullock et al. 2013). The point estimates generated using ADBOU were used in the hazards analysis described below. For individuals with unfused or fusing epiphyses, age‐at‐death was estimated using epiphyseal fusion and/or dental development (Buikstra and Ubelaker 1994; Liversidge and Marsden 2010; Scheuer and Black 2004; Smith 1991). All individuals aged 15+ years were included in this study.

### 2.2 Sex Estimation

Sex was estimated using morphological and metric traits of the pelvis, cranium, and long bones. Morphological elements of the cranium (glabella, orbital margin, nuchal crest) and pelvis (subpubic contour, ventral arc, medial aspect of the ischio‐pubic ramus) were scored using the Morphological pelvis and skull sex estimation (MorphoPASSE) scoring guidelines (Klales and Cole 2018; Klales et al. 2012; Walker 2008). The MorphoPASSE database was used to classify individuals using the flexible machine learning statistical option (random forest modeling), which is a nonparametric technique that increases classification accuracy with fewer statistical assumptions than regression models (Klales et al. 2020). The output for this approach provides the posterior probability that an individual is a member of a certain sex category. Post‐cranial measurements of the humerus (epicondylar breadth, maximum length, and vertical and transverse head diameter) and femur (vertical head diameter and bicondylar width) were also taken for all individuals with fused long bone epiphyses (Buikstra and Ubelaker 1994). Metric sex categories were defined using the sample‐specific mean as a sectioning point. An individual was classified as male if the measurement was greater than the mean and female if the measurement was equal to or less than the mean (Albanese et al. 2005; Spradley and Jantz 2011). The femoral and humeral scores were combined to create a single metric sex classification. At worst, this sample‐specific approach underestimates any sex‐related effects in the data. The post‐cranial sex estimate was prioritized in instances where the morphological and metric estimates did not align and only cranial morphological traits were scored. This approach is based on evidence that sex estimates from post‐cranial elements outperform those of the skull, and thus should be used when the pelvis is unavailable (Spradley and Jantz 2011).

Individuals were classified as male or female and coded as 0 or 1, respectively. It is recognized that the categories of “male” and “female” are not reflective of the diverse physiological, psychological, and social experiences of all individuals (Astorino 2019; DuBois and Shattuck‐Heidorn 2021; Vora and Srinivasan 2020). Ultimately, the dichotomization of the sex variable here is an attempt to capture the genetic and hormonal variation known to affect sex‐specific mortality risks in living populations.

### 2.3 Hazards Model

Hazards analysis was used to examine the effect of sex on mortality risks. Fully parametric models can accommodate small sample sizes typical of bioarchaeological data and can smooth random variation in the mortality profile without imposing a particular age pattern (Gage 1988; Wood et al. 2002). The Gompertz model is a parsimonious model of senescent mortality (Gage 1991; Wood et al. 2002):

$$h\left(t\right)=\alpha e^{\mathrm{\beta t}}$$

where *t* is age, α represents the overall level of mortality due to aging, and β represents the rate at which the risk of mortality increases with age. The Gompertz‐Makeham model adds a third parameter, $\alpha_{1}$, which represents the constant age‐independent risk of adult mortality (Gage 1988; Makeham 1860; Wood et al. 2002).

$$h\left(a\right)=\alpha_{1}+\alpha_{2}e^{\mathrm{\beta a}}$$

Preliminary analysis of the data revealed that the addition of the age‐independent component improved the fit of the model. Therefore, the three‐parameter Gompertz‐Makeham model was selected, and sex was modeled as a covariate directly on the baseline hazard using a proportional hazards specification:

$$h_{i}\left(t_{i}|x_{i}\rho \right)=h\left(t_{i}\right)e^{\left(x_{i}\rho \right)}$$

where the baseline hazard *h*(*t*_*i*_) = $\alpha_{1}+\alpha_{2}e^{\mathrm{\beta a}}$, *t*_*i*_ is the age of the *i*th person in years, *x*_*i*_ is the sex covariate, and *ρ* is the parameter representing the effect of the covariate on the baseline hazard.

The Gompertz‐Makeham parameters can be estimated with samples of less than 100 (El‐Sherpieny et al. 2013), and all model parameters were estimated using maximum likelihood analysis in the *mle* program (Holman 2003). Estimated sex was recorded as 0 (male) or 1 (female), and a negative or positive estimate for the parameter representing the effect of the covariate on the hazard suggests that the female sex category is associated with lower or higher risks of mortality, respectively. A likelihood ratio test (LRT) was used to evaluate whether the addition of the covariate improved the fit of the model (i.e., *H*_0_: effect of the covariate = 0). The LRT is a simple method for comparing alternative models and is computed as follows: −2 [ln(L_sex_) − ln(L_baseline_)], where LRT approximates a $\chi^{2}$ distribution with a df = 1 (Hilborn and Mangel 1997, 154). The 95% confidence intervals for the estimated effects of the sex covariate between the (1) early medieval and (2) late medieval period were used to assess variability in the degree of sex differences in mortality risks across time periods. A higher or lower sex covariate parameter in the late medieval period suggests a respective increase or decrease in female mortality risks, if the confidence intervals do not include zero. Overlapping confidence intervals between the two time periods was interpreted as failing to indicate a significant difference, rather than indicating no difference. Significance was assessed at α = 0.1 for all statistical analyses.

## 3 Results

Irish females were expected to have higher mortality risks compared to males, but as seen in Table 2 the results of the Gompertz‐Makeham hazards model failed to demonstrate a difference in mortality risks based on the sex variable. While the estimated value of the parameter representing the effect of the sex covariate is positive (i.e., suggesting higher female mortality risks), these results cannot be interpreted as reflecting a significant difference. The confidence interval spans zero and the results of the LRT revealed that the addition of the sex covariate did not improve the fit of the baseline hazard model. The confidence intervals for the estimated effect of the sex covariate for the early medieval and late medieval periods also span zero and overlap one another, which suggests that the effect of the sex covariate on the model did not significantly vary across time periods. Overall, these results fail to reveal an effect of sex on mortality risks in medieval Ireland and no significant variation was observed over time.

**TABLE 2:** Maximum likelihood estimates of the effect of the sex covariate (0 = male, 1 = female) on mortality risks, the likelihood ratio test results for the Gompertz‐Makeham model, and the 95% confidence intervals for the estimated effects of the sex covariate across time periods.

	Effect of sex covariate (s.e.)	Confidence interval	−2LLR (*p*)
All individuals (*n* = 335)	0.07 (0.11)	−0.18, 10.0	0.51 (0.47)
Early medieval (*n* = 97)	0.01 (0.24)	−0.51, 0.43	—
Late medieval (*n* = 238)	0.03 (0.12)	−0.27, 0.31	—

## 4 Discussion

### 4.1 Effect of Sex on Mortality Risks

**FIGURE 2:**
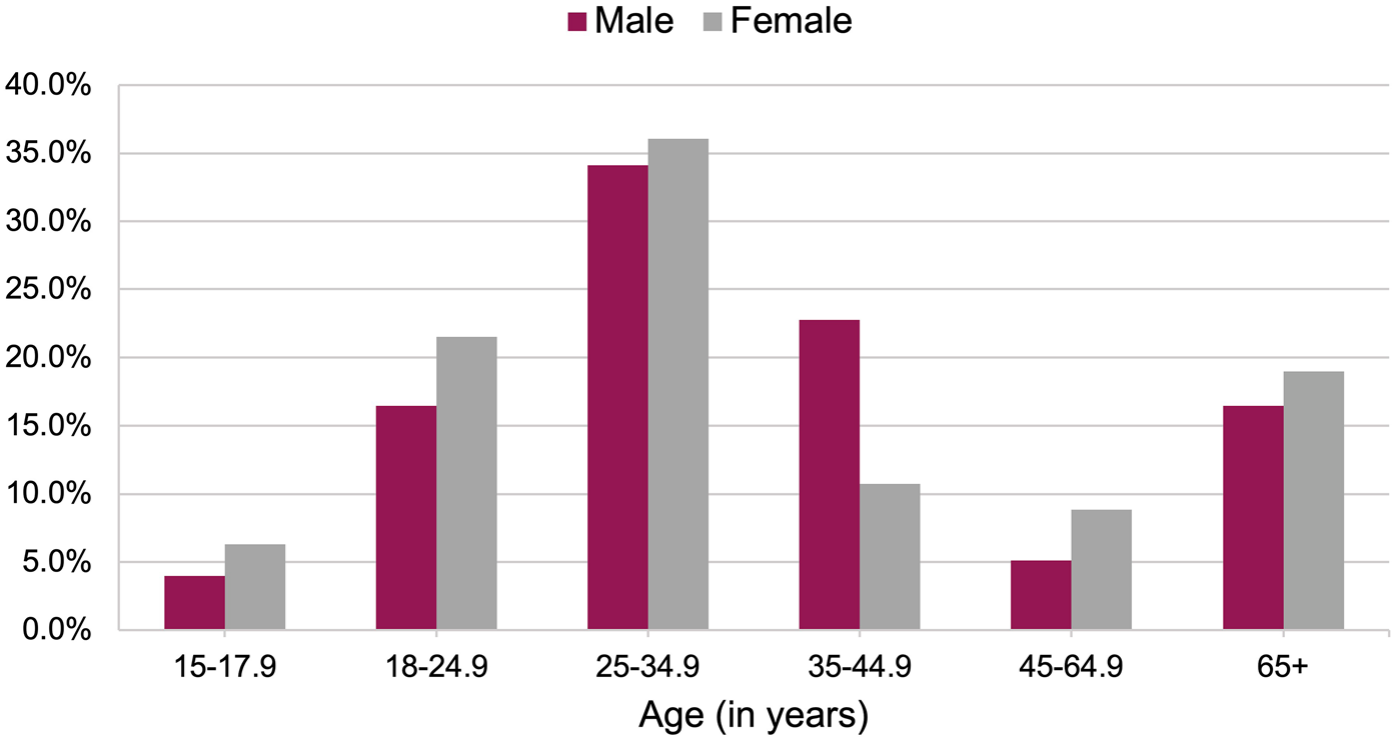
Sex‐specific age‐at‐death distributions.

A widespread female mortality disadvantage has been documented across medieval Europe, with an estimated 2.6 to 3.9 million females unaccounted for in the historical and archaeological record (Bardsley 2014). Several factors have been proposed to explain these “missing females” in the medieval period, including a cultural preference for males, nutritional and environmental differences, and maternal mortality (Bardsley 2014; Bullough and Campbell 1980). As noted above, the results of this study failed to demonstrate a female mortality disadvantage in the context of medieval Ireland. Nevertheless, the similar mortality profiles for Irish males and females, seen in Figure 2, could reflect the presence of cultural barriers and/or differential environmental exposures that counteracted the innate female survival advantage. In other words, if Irish men and women were treated equally and experienced similar living conditions, it would be expected that females would have lower mortality risks compared to males. Thus, the departure from this trend suggests that the biological mechanisms tied to the female survival advantage were unable to overcome the social and environmental circumstances of medieval Irish women. The following discussion explores the social, environmental, and methodological factors that could be influencing the results of this study.

Medieval Irish society was a patriarchal hierarchy, where an individual's social identity was primarily determined by their gender and social status. Irish women were generally considered to be physically, intellectually, emotionally, and spiritually inferior to men (Bitel 1992, 1996). A cultural male preference was deeply‐rooted in the sociopolitical structure of Irish society, as women were unable to formally wield political power, restricted in their legal rights (e.g., the ability to inherit land), and had limited autonomy compared to their male counterparts (Kelly 1988; Kenny 2013; Oxenham 2016). Despite the widespread evidence that patriarchal societal structures are deleterious to female health and survival (Beltrán Tapia and Gallego‐Martínez 2017; Pennington et al. 2023), no sex‐based differences in mortality risks were observed in this study. The failure to detect an effect of sex on mortality risks could reflect the hierarchical nature of medieval Irish society. Irish women were not solely grouped by their shared sex, but by their social, marital, and legal status, possessions and professions as well as their religious or secular status, and thus were not unequivocally at the bottom of the social hierarchy (Oxenham 2016). Moreover, modern patriarchal social structures with a high degree of gender inequity are also associated with increased levels of male excess mortality (particularly for lower status males) (Kavanagh et al. 2017; Kruger et al. 2014; Stanistreet 2005). This association is partially due to harmful male gendered behaviors, such as resource competition and guarding that lead to warfare and interpersonal conflict (Kruger et al. 2014). Hence, the patriarchal hierarchal structure of Irish society could have produced differential mortality risks based on an individual's social status, not solely shared sex. Future research will explore the interaction of social status and sex in this context, as recent bioarchaeological research has shown the intersectional effects of these aspects of social identity on health and mortality (Yaussy 2019, 2024).

Female‐biased mortality has been connected to sex‐based differences in nutritional intake across medieval Europe (Baldoni et al. 2021; Herold 2008; Šlaus 2000), and the documentary evidence from medieval Ireland outlines clear status‐ and gender‐based differences in nutritional resource accessibility and allotment (Kelly 1988). Individuals of shared social status had access to certain types of cereals and other food products, and women received the same foods as their husbands but in smaller portions (note: little documentation is available on the diet of unmarried Irish women) (McClatchie et al. 2015; Peters 2015). The archaeological and isotopic evidence from medieval Ireland reflects a diet comprised of plant‐based foods supplemented with terrestrial animal protein (McClatchie et al. 2015; McCormick et al. 2014; McKenzie et al. 2020; Ryan et al. 2018). A study on dental pathologies found a higher rate of dental caries and a lower frequency of dental calculus in Irish females compared to males across several early medieval sites, which could suggest females consumed less animal protein and more carbohydrates (Novak 2015). However, diet is only one factor that affects sex‐based patterns of dental pathologies; others include differences in dental development, steroid hormones, and a genetic predisposition related to the X chromosome (Lipsky et al. 2021; Lukacs and Largaespada 2006). Moreover, a recent isotopic study found no differences in diet based on sex and little variation across the medieval period at a site in western Ireland (McKenzie et al. 2020). Additionally, the bioarchaeological evidence demonstrates a high frequency of nutritional and metabolic stressors (e.g., cribra orbitalia, linear enamel hypoplasia) throughout medieval Ireland, regardless of sex (Manifold 2014; McKenzie and Murphy 2018; Novak 2015; Novak et al. 2017; Scott 2017; Tesorieri 2012, 2016). This parallels the finding of Godde and Hens (2021) that males and females with cribra orbitalia had similar risks of dying in medieval London, which suggests that iron deficiency and parasitic infections were endemic during this period. Thus, the similarities observed in the mortality profile here could reflect widespread food insecurity and insufficient nutrients in this context.

Another possible explanation for the inability to detect an effect of the sex covariate on the model could relate to regional heterogeneity in sex‐specific mortality risks. Nutritional and metabolic non‐specific skeletal stress indicators have been found to vary regionally across early medieval sites, with areas of reduced land use capability suffering poorer health (Scott 2017; Tesorieri 2016). The southern and eastern regions of Ireland were well‐suited for the cultivation of cereals–due to their arable land and being sheltered from oceanic climatic conditions. However, the western and northern regions in the medieval period were prone to food shortages and insecurity, due to being dominated by poorly drained wetlands and strong winds and rains from the Atlantic Ocean (Clarke 2009; Haughton 2008). Historic demographic data from the last few centuries suggest that females generally live longer than males in populations exposed to famine and epidemic conditions (Zarulli et al. 2018). Thus, Irish females could have been more buffered than their male counterparts in regions with increased food insecurity. However, Tesorieri (2016) found that in the northern region–an area largely unsuitable for agricultural purposes (Haughton 2008)–fewer females lived past 25 years of age compared to their male counterparts. Additionally, in the proximate context of medieval London, no female bias in survivorship has been found in famine settings (DeWitte and Yaussy 2020; Yaussy et al. 2016). In fact, DeWitte and Yaussy (2020) found that males experienced lower risks of mortality in famine settings during the 14th–16th centuries. It was outside the scope of this study to evaluate regional variation in the effect of sex on mortality risks, but future research will explore this relationship, as regional and geographic heterogeneity in food security likely had sex‐specific effects on mortality in Ireland.

Several bioarchaeological studies on pre‐industrial contexts have lent support to a survival disadvantage for females of reproductive age (Fojas 2022; Wilson 2014), which is commonly attributed to high rates of fertility and maternal mortality in the past. However, bioarchaeological evidence of maternal mortality in medieval Ireland is sparse. In an analysis of 15 medieval Irish sites, Murphy (2021) identified 29 probable maternal deaths (i.e., an adult female burial with at least one fetal/perinatal infant) out of 1759 excavated burials. This rate of 1.7% is likely a gross underestimation of the true figure, as the World Health Organization estimated in 2017 that 9.2% of reproductive‐aged female deaths (15–49 years) were the result of maternal causes—down from 26.3% in 2000 (World Health Organization 2019). Irish reproductive‐aged females also faced increased susceptibility to infection, due to the effect of hormonal alterations during pregnancy on immunocompetency (Chan and Smith 2018; Kourtis et al. 2014). However, pregnant Irish women were afforded cultural protections, such as increased food allotments and exceptions to normal practices of nutritional intake (Ní Chonaill 2008; Peters 2015), that could have buffered them against this period of reduced immunocompetence. It also could have protected them against iron and protein deficiencies that are hypothesized to have had deleterious effects on female survival in other medieval contexts (Bardsley 2014; Bullough and Campbell 1980; Herold 2008). As seen in Figure 2, the female age‐at‐death distribution does peak in reproductive age categories (18–24.9 and 25–34.9 years), which could suggest pregnancy and childbirth as a principal contributor to female mortality risks in Ireland. However, there is a need to look beyond maternal mortality to understand patterns of female health and mortality in the past, as even non‐reproducing medieval settings (e.g., nunneries) have female age‐at‐death distributions that peak during reproductive ages (16–45 years) (Podd 2020). Further research on the health status of reproductive‐aged females is needed to gain insight into sex‐specific risks of mortality during the medieval period in Ireland (McFadden and Oxenham 2019; Riccomi et al. 2021).

As aforementioned, females today are less susceptible to most infections due to an innate immunological advantage (Chlamydas et al. 2022) and are more resilient during high mortality events, including epidemics (Zarulli et al. 2018). However, both DeWitte (2009) and Godde et al. (2020) found that males and females were similarly susceptible to the Black Death (c. 1349–1350 ce) in medieval London. Conversely, a female bias in mortality was found in the Netherlands during the Black Death (c. 1349–1351 ce) and in the subsequent recurring years of plague (Curtis 2021; Curtis and Roosen 2017). This variability in sex‐specific mortality patterns during epidemic events could suggest context‐specific differences in risks of pathogen exposure between males and females across medieval Europe. Several studies have demonstrated that females historically had increased exposure to infectious pathogens during early childhood, adolescence, and reproductive‐aged years, due to cultural norms of gendered behavior such as childcare responsibilities, indoor domestic labor roles, and dietary restrictions (Goldin and Lleras‐Muney 2019; Shapland et al. 2015; Tabutin and Willems 1998). Irish children were raised and educated according to their gender and social rank, and women participated in tasks in both agricultural and domestic spheres, including textile production, dairying, and childbearing and rearing (Bitel 1996; Ní Chonaill 1997, 2008; Valante 2022). Thus, it is possible that the results reflect higher risks of pathogen exposure for Irish females, due to indoor domestic labor roles and frequent interaction with livestock. However, no clear age‐ or sex‐based patterns of specific (e.g., tuberculosis) or non‐specific skeletal markers of infection (e.g., periosteal new bone formation) have been found in the medieval Irish bioarchaeological record (McKenzie and Murphy 2018; Power 1997; Tesorieri 2012).

Gompertz‐Makeham hazards models have successfully been used with the proportional hazards specification in bioarchaeological research to reveal sex‐based differences in the medieval period (e.g., DeWitte and Yaussy 2020; Yaussy et al. 2016). The benefits of this approach are its ability to accommodate small sample sizes, minimize the number of parameters in the model, and smooth random variation without imposing a particular fixed age pattern on the data (Gage 1988; Wood et al. 2002). However, one limitation is its inability to detect age‐related patterns. The modeling of the sex covariate using this specification cannot capture age‐specific differences in mortality risks, because it assumes that the effect of the covariate on the baseline hazard stays proportional over time, that is, is independent of age. Age‐specific differences in mortality risks between males and females play a critical role in shaping sex differentials in mortality (Vaupel et al. 2021; Zarulli et al. 2021). Thus, the failure to detect an effect of the sex covariate here could relate to undetected differences in age‐specific mortality risks between Irish males and females. Ultimately, further analysis is needed to test whether the effect of the sex covariate varied across age groups in this context.

Additionally, the use of the binary sex variable in this study may have unintentionally biased the results. Human sexual variation is produced by a complex interaction of genetic, hormonal, and chromosomal processes resulting in the differential expression of phenotypic traits (e.g., external/internal genitalia, secondary sexual characteristics) that are used to classify individuals as male, female, or intersex (DuBois and Shattuck‐Heidorn 2021; Rioux et al. 2022; Zuckerman and Crandall 2019). In bioarchaeological contexts, sex is most commonly estimated using morphometric traits of the skull and pelvis that are shaped by the secretion of gonadal steroid hormones during puberty (e.g., estrogens, androgens) (Almeida et al. 2017; Fitzpatrick 2004). However, hormone levels have a high degree of individual‐level variation and fluctuate over the life course (Cameron 2003), and genetic and environmental factors affect the expression of sexually disparate skeletal traits across populations (Ubelaker and DeGaglia 2017). Hence, our ability to capture the spectrum of sexual variation observed in living populations is constrained in past contexts. Moreover, the reliance on a binary categorization system for sex estimation results in the exclusion of individuals lacking a confident male/female assignment. It is important to recognize that this inability to estimate sex reflects the scorer's level of certainty, not the true spectrum of human sexual variation (Geller 2005; Wesp 2017). Additionally, sex and gender were essentially collapsed into a single category for the purposes of statistical analysis, which can confound the influence of other biosocial and environmental factors on mortality risks (Zuckerman et al. 2023). Hence, the results of this study could reflect the heterogeneity contained in the binary sex variable and may not accurately reflect the reality of sex‐specific mortality risks in medieval Ireland. As explored above, several other biosocial and environmental factors, including social status, age, and unique regional pressures, could have contributed to the production of differential mortality risks in this context. Future research will continue to work towards a more nuanced understanding of the drivers of mortality risks in medieval Ireland by exploring how sex intersected with the biosocial and environmental elements discussed above.

### 4.2 Temporal Variation in Mortality Risks

The Medieval Climate Anomaly (MCA) spanned the mid‐10th to mid‐13th centuries and was marked by increased temperatures and decreased precipitation in Europe (Trouet et al. 2009). The MCA transition to the Little Ice Age (LIA) began in the mid‐13th century and brought a sharp decline in temperatures as well as an increase in precipitation (Büntgen et al. 2011; Trouet et al. 2009; Turney et al. 2006) that produced harvest‐related crises, food insecurity, and famine conditions across Europe (Campbell and Ludlow 2020). In the beginning of the 14th century, Irish communities began to feel the effects of this period of climatic instability through repeated famines and food shortages and mortality crises (e.g., the Great European Famine c. 1315–18 ce, the Black Death c. 1348–49 ce) (Campbell and Ludlow 2020; Kelly 2004; Murphy 2018). Several bioarchaeological studies on medieval London (c. 1000–1540 ce) have found sex‐specific trends in mortality risks that overlap this tumultuous period in history. For example, no sex‐based differences in mortality risks were found in adults during the pre‐Black Death period (c. 1000–1250 ce) (DeWitte and Yaussy 2020; Yaussy et al. 2016), but adult males experienced lower risks of mortality compared to females in the post‐Black Death period (c. 1350–1540 ce) (DeWitte and Yaussy 2020). The evidence from Ireland is more limited, but there is data to support a similar sex‐specific temporal trend in mortality during the late medieval period. At Bakehouse Lane in Co. Waterford, Power (1997) found an increase in mean age‐at‐death for all individuals from the mid‐11th to the early‐12th century. However, mean age‐at‐death declined from the early‐12th to the mid‐13th–16th centuries for Irish females, but increased for males. Notably, this decline in female age‐at‐death occurred during the abovementioned period of climatic instability, mortality crises, and stagnant economic growth in Ireland (Campbell 2016; Campbell and Ludlow 2020).

Given the abovementioned evidence, it was expected that the effect of the sex covariate would vary across time periods with female mortality risks increasing from the early to the late medieval period in Ireland. However, as seen in Table 2, no temporal variation in the sex covariate was observed in the model. In a recent study, Clark et al. (2024) found no change in the age‐at‐death distribution from the late medieval to the post‐medieval (c. 1550–1800) period in sites located in the English Pale (a.k.a., the counties of Dublin, Meath, Kildare, and Louth) when all individuals were considered together. However, when males and females were analyzed separately, the median age‐at‐death for males increased over time, but not for females. Thus, dividing the sample by sex category prior to analyzing temporal variation in mortality risks could reveal differences between Irish males and females not observed here. Clark et al. (2024) also found that both female and male survivorship varied significantly across late medieval sites. This echoes the aforementioned notion that the failure to detect an effect of the sex covariate on the model could reflect the confounding effects of regional and geographic heterogeneity.

As discussed above, Irish females could have been at increased risk of pathogen exposure due to social and biological factors, including indoor domestic labor roles and frequent interaction with livestock. Thus, increased urbanization and population density during the late medieval period, particularly in the southern and eastern regions of the island, could have had a disproportionate effect on female mortality risks. The population of Ireland is estimated to have increased from around half a million people in the early medieval period to as high as 1.4 million by the late 13th century (Gillespie 2007). While these figures are speculative, the dendrochronological data support an increase in population size and density between the 12th–14th centuries (Campbell and Ludlow 2020; Hall 2003; Murphy 2018). Walter and DeWitte (2017) found that females living in urban settings experienced increased mortality risks compared to their rural counterparts in medieval England, whereas mortality risks were similar for males across geographic environments. Hence, the inability to detect a temporal shift in female mortality risks could reflect differences between urban and rural Irish communities. In Ireland, individuals living in towns were more buffered during the abovementioned periods of food shortages and insecurities compared to their rural counterparts (see Galloway and Murphy 2023), and thus could have experienced lower mortality risks in the late medieval period. Moreover, demographic data from the 19th and 20th centuries suggest that Irish rural women were more vulnerable to tuberculosis compared to their male counterparts, but this sex difference was negligible in urban settings (Jones 2001). Thus, the relationship between mortality risks and urban and rural contexts in medieval Ireland could have been reversed from what was observed by Walter and DeWitte (2017). Ultimately, further investigation is needed to understand how sex‐specific mortality risks fluctuated during the early and late medieval periods and how this varied across Irish communities in different geographic and regional settings.

## 5 Conclusion

The results of this study failed to document an effect of sex on mortality risks in medieval Ireland (c. 500–1550 ce). Additionally, no variation in the degree of sex differences in mortality risks was observed between the early (500–1150 ce) and late medieval (1150–1550 ce) periods. The similarity in the mortality profiles of males and females is likely due to mortality risks being high for all Irish individuals, regardless of sex, during a period marked by foreign invasions, prolonged periods of warfare, shifts in ethnic and religious identity, and mortality crises, including epidemic and famine events. However, the lack of a distinct female survival advantage in this context could suggest the presence of cultural barriers and/or differential environmental exposures that disproportionately affected female health and mortality. Future research will continue to explore the other biosocial and environmental factors that could have been influencing sex‐specific mortality risks in this context.

## Author Contributions

**Allison C. Ham:** conceptualization (lead), data curation (lead), formal analysis (lead), funding acquisition (lead), investigation (lead), methodology (lead), project administration (lead), resources (lead), software (lead), supervision (lead), validation (lead), visualization (lead), writing – original draft (lead), writing – review and editing (lead).

## Data Availability

The data that support the findings of this study are available from the corresponding author upon reasonable request.
